# Epidural bolus versus continuous epidural infusion analgesia on optic nerve sheath diameter in paediatric patients: A prospective, double-blind, randomised trial

**DOI:** 10.1038/s41598-020-62273-8

**Published:** 2020-03-25

**Authors:** Bora Lee, Jae Hoon Lee, Min-Soo Kim, Seon Ju Kim, Jeehyun Song, Do-Hyeong Kim, Yong Seon Choi

**Affiliations:** 1 Department of Anaesthesiology and Pain Medicine, Severance Hospital and Anaesthesia and Pain Research Institute, Yonsei University College of Medicine, 50-1 Yonsei-ro, Seodaemun-Gu, Seoul 03722 Republic of Korea; 2 Department of Anaesthesiology and Pain Medicine, Anaesthesia and Pain Research Institute, Gangnam Severance Hospital, Yonsei University College of Medicine, 211 Eonju-ro, Gangnam-gu, Seoul 06273 Republic of Korea

**Keywords:** Paediatric research, Paediatric research

## Abstract

The use of programmed intermittent epidural bolus for postoperative analgesia may have greater analgesic efficacy than continuous epidural infusion. However, the rapid delivery speed used with an epidural bolus is more likely to increase intracranial pressure. We compared the effects of lumbar epidural bolus versus continuous infusion epidural analgesia on intracranial pressure in children using optic nerve sheath diameter as a marker. We randomly assigned 40 paediatric patients to bolus or infusion groups. Epidural analgesia (0.15% ropivacaine 0.3 ml·kg^−1^) was administered via bolus or infusion. Ultrasonography was used to measure the optic nerve sheath diameter before (T0), at 3 min (T1), 10 min (T2), and 70 min (T3) after starting the pump. There were statistically significant between-group differences in optic nerve sheath diameter over time (P_Group x Time_ = 0.045). From T0–T3, the area under the curve values were similar between the two groups. Although there were differences in the patterns of optic nerve sheath diameter change according to the delivery mode, the use of lumbar epidural bolus did not increase the risk of intracranial pressure increase over that of continuous infusion. Further research is needed to investigate intracranial pressure changes after continuous application of each delivery mode.

## Introduction

Inadequate use of analgesia after an operation causes unacceptable pain, generates long-lasting pain memories, and increases the risk of behavioural disorders in paediatric patients^1,2^. When possible, regional analgesia should be performed to optimise postoperative pain management^3^. Continuous epidural catheterisation under general anaesthesia has been used to provide sufficient postoperative analgesia in the days following surgery^3,4^, and epidural analgesia is effective for postoperative pain control in paediatric patients^5–8^.

Compared with continuous epidural infusion, programmed intermittent epidural bolus can have greater analgesic efficacy and fewer side effects in surgical patients^9–11^. However, an epidural bolus may generate relatively higher injection pressure because the delivery speed of a solution through the epidural catheter is directly related to peak pressures^12^. Thus, compared with continuous epidural infusion, pressure changes after programmed intermittent epidural bolus use are more likely to increase epidural pressure and, subsequently, intracranial pressure. Use of caudal block causes an increase in intracranial pressure in paediatric patients^13^, but the effect of epidural analgesia on intracranial pressure in paediatric patients has not been investigated. Studies of non-invasive methods used for intracranial pressure measurement have demonstrated that optic nerve sheath diameter is correlated with intracranial pressure; optic nerve sheath diameter measurement has high diagnostic accuracy for detection of increased intracranial pressure in children^14–16^. This prospective, randomised, double-blinded study aimed to investigate the effects of lumbar epidural bolus and infusion on intracranial pressure using ultrasonographic optic nerve sheath diameter measurements in children.

## Results

**Figure 1:**
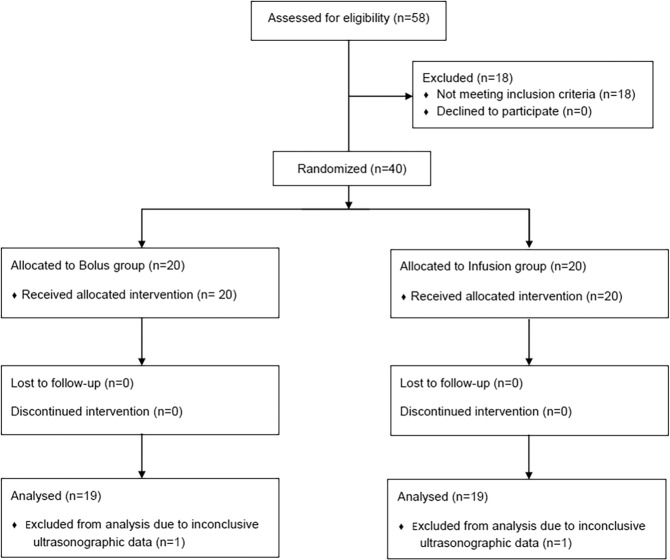
Flow diagram of the study.

Forty of the 58 patients assessed for eligibility were enrolled in the study (August 2017 to May 2018) and were randomly assigned to either the epidural bolus or infusion group. Thirty-eight of the 40 patients completed the study (Fig. 1). Two subjects were excluded from the final analysis because optic nerve sheath borders were not apparent at the follow-up ultrasonographic measurement. The mean value of T1–T0 was 10 min. Patient characteristics and intraoperative variables were similar between the two groups (Table 1).

**Table 1:** Patient characteristics and operative data in the lumbar epidural bolus and infusion group.

	Bolus group (n = 19)	Infusion group (n = 19)	P value
Age (years)	9.42 ± 2.95	9.24 ± 2.62	0.840
Sex (male)	12 (63)	13 (68)	>0.999
Height (cm)	128.4 ± 16.7	123.0 ± 17.7	0.348
Weight (kg)	29.5 ± 9.2	26.5 ± 8.5	0.302
Body mass index (kg·m^−2^)	17.4 ± 3.0	17.5 ± 4.1	0.898
Anaesthesia time (min)	306 ± 119	346 ± 105	0.278
Operation time (min)	240 ± 108	290 ± 98	0.144
Recovery time (min)	50.0 (42.5–59.0)	42.0 (40.0–52.5)	0.395

Values are presented as mean ± SD, median (IQR), or number (proportion).

Table 2 presents the results of analyses of hemodynamic data and data for variables potentially affecting intracranial pressure (e.g., end-tidal carbon dioxide concentration, end-tidal sevoflurane concentration, and peak inspiratory pressure). There were no statistically significant between-group differences among these variables at any time point.

**Table 2:** Hemodynamic data and parameters associated with intracranial pressure at each time point in the lumbar epidural bolus and infusion groups.

	Bolus group (n = 19)	Infusion group (n = 19)	P value
**Heart rate (beats.min**^**−1**^**)**			
T0	110 ± 18	109 ± 18	0.905
T1	103 ± 16	97 ± 17	0.269
T2	95 ± 18	92 ± 18	0.634
T3	97 ± 19	100 ± 17	0.618
**Mean arterial pressure (mmHg)**			
T0	75.2 ± 9.9	75.0 ± 11.9	0.941
T1	73.3 ± 12.5	72.7 ± 11.0	0.880
T2	69.0 (66–78)	77.0 (69–80)	0.267
T3	75.0 ± 7.8	75.8 ± 8.7	0.755
**Peak inspiratory pressure (cmH**_**2**_**O)**			
T0	13.1 ± 1.7	12.0 ± 1.9	0.080
T1	12.7 ± 1.7	11.7 ± 2.2	0.147
T2	12.8 ± 1.7	11.8 ± 2.1	0.117
T3	13.5 ± 2.2	12.8 ± 2.6	0.346
**End-tidal carbon dioxide (mmHg)**			
T0	34.4 ± 1.2	33.8 ± 1.6	0.220
T1	34.3 ± 1.9	33.8 ± 1.3	0.437
T2	33.7 ± 1.8	34.2 ± 1.8	0.482
T3	34.0 (33.0–35.5)	34.0 (33.0–35.5)	0.688
**End-tidal sevoflurane concentration (%)**			
T0	1.57 ± 0.45	1.41 ± 0.36	0.226
T1	1.40 (1.20–1.65)	1.20 (1.10–1.65)	0.171
T2	1.6 (1.25–1.90)	1.60 (1.30–1.70)	0.758
T3	2.20 ± 0.28	2.37 ± 0.35	0.100

Values are presented as mean ± SD or median (IQR).

**Figure 2:**
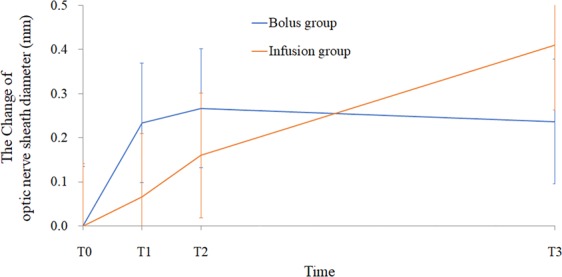
Changes in optic nerve sheath diameter. Values are expressed as estimated mean ± standard error values from linear mixed model. *The linear mixed model revealed that there were statistically significant between-group differences in changes in ONSD according to time (model corrected for age, gender, height, weight, end-tidal sevoflurane and carbon dioxide levels, and peak inspiratory pressure; P_Group × Time_ = 0.045). T0, before starting the pump; T1, 3 min after starting the pump; T2, 10 min after starting the pump; T3, 70 min after starting the pump.

Table 3 presents the mean values for optic nerve sheath diameter from the linear mixed model and the corresponding P values. The analysis revealed statistically significant between-group differences for changes in optic nerve sheath diameter (P_Group × Time_ = 0.045) (Fig. 2). The post-hoc analysis revealed that optic nerve sheath diameter at T2 was significantly increased from baseline in the bolus group and that optic nerve sheath diameter at T3 was significantly increased from baseline in the infusion group. Although the patterns of increase in optic nerve sheath diameter differed between the two groups, the maximal increase in optic nerve sheath diameter from baseline was not statistically significant between the groups (bolus group: 0.27; infusion group: 0.41; P > 0.999). The areas under the curve for T0–T3 were similar for the two groups.

**Table 3:** Changes in optic nerve sheath diameter between time points in the lumbar epidural bolus and infusion group.

	Bolus group (n = 19)	Infusion group (n = 19)	P value
**Optic nerve sheath diameter (mm)**			
T0	5.33 ± 0.14	5.25 ± 0.14	0.674
T1	5.56 ± 0.13	5.31 ± 0.14	0.200
T2	5.60 ± 0.13	5.41 ± 0.14	0.330
T3	5.57 ± 0.14	5.66 ± 0.15	0.633
Changes in optic nerve sheath diameter (mm)			**Adjusted P value**
T1-T0	0.23 ± 0.09	0.07 ± 0.09	>0.999
T2-T0	0.27 ± 0.09*	0.16 ± 0.09	>0.999
T3-T0	0.24 ± 0.1	0.41 ± 0.11*	>0.999
T2-T1	0.03 ± 0.09	0.09 ± 0.09	>0.999
T3-T1	0 ± 0.1	0.34 ± 0.12 *	0.0498
T3-T2	−0.03 ± 0.1	0.25 ± 0.11	>0.999

Values are presented as estimated mean ± standard error from the linear mixed model. Adjusted P value indicates the Bonferroni-corrected P value. *Adjusted *P* < 0.05 in each group.

## Discussion

This study is the first to investigate the effects of lumbar epidural analgesia on intracranial pressure comparing the use of bolus and infusion protocols in paediatric patients. Our results revealed a faster increase of optic nerve sheath diameter in the bolus group compared with the infusion group. Although there were differences in the patterns of optic nerve sheath diameter change according to the method of injection, the total optic nerve sheath diameter changes were not significantly different between the two groups.

The prevalence of moderate-to-severe pain in paediatric patients remains high^17^. Use of regional analgesia may play a major role in pain score reduction^3^. Epidural patient-controlled analgesia can be used for paediatric patients in major thoracic, abdominal, or orthopaedic surgeries^3,4^. In particular, effective epidural analgesia prevents muscle spasms that are primarily a spinal reflex caused by pain in children with cerebral palsy who undergo extensive lower limb orthopaedic surgery^8^. Epidural injection provides analgesia via the spread of local anaesthetic through the noncontinuous epidural space. The distribution of local anaesthetics into the epidural space is not uniform and is directed into multiple parallel passages^18^. A more uniform and extensive spread occurs when large volumes and high injectable pressures are administered^18,19^. With the development of patient-controlled analgesia units, the injection mode can be controlled using programmed intermittent epidural bolus or continuous epidural infusion^20^. After posterior spinal fusion for adolescent idiopathic scoliosis, cumulative opioid consumption is lower when the intermittent bolus function is used than with the continuous infusion mode; rates of postoperative nausea, vomiting and pruritus are much greater when the continuous infusion mode is used^11^. Use of programmed intermittent epidural bolus reduces anaesthetic consumption during labour analgesia and decreases labour pain and motor block^9,10,21,22^. However, bolus injection into a confined space may increase epidural pressure and intracranial pressure, especially in children.

The variation in intracranial pressure correlates with the optic nerve sheath diameter because the optic nerve is surrounded by distensible subarachnoid space^23^. Hansen *et al*. found that changes in optic nerve sheath diameter are closely associated with intracranial pressure variations within a limited intracranial pressure interval (20–50 mmHg), and ongoing enlargement of optic nerve sheath diameter on serial ultrasonography has clinical relevance^24^. Several studies of the relationship between optic nerve sheath diameter and intracranial pressure have shown that optic nerve sheath diameter is a useful method for detecting elevated intracranial pressure in children and adults^15,25–31^.

The optic nerve sheath diameters were compared before starting the pump, and 3 min, 10 min, and 70 min after starting the pump between the single bolus and continuous infusion protocols outlined in this study. The second optic nerve sheath diameters were measured at 3 min after the pump was started because it took up to 3 min for the bolus injection to reach completion in the bolus group. Previous studies have found that optic nerve sheath diameter increases to a peak value approximately 10 min after an increase in epidural pressure and intracranial pressure^13^. Based on this, optic nerve sheath diameter was measured at the times of 10 min after starting the pump in the bolus group and 10 min after completion of continuous infusion in the infusion group. In the bolus group, as we expected, the maximum value was observed 10 min after the epidural bolus was administered, rather than immediately afterward. We also expected that the lumbar epidural bolus would result in a greater increase in optic nerve sheath diameter than would continuous infusion because the delivery speed was much faster in the bolus group^12^. However, although the optic nerve sheath diameter rapidly increased in the bolus group, epidural bolus did not cause a greater increase in optic nerve sheath diameter than did infusion. There were continuous increases in the optic nerve sheath diameter in the infusion group for 70 min that were similar in degree to those observed in the bolus group. The total burdens of optic nerve sheath diameter (areas under the curve) were not significantly different between the two groups. This result suggests that the risks of intracranial pressure increases were similar between groups and that the bolus group was not associated with a greater risk of intracranial pressure increase than was the infusion group. The rates of nausea and vomiting are higher during the postoperative period among patients exposed to continuous epidural infusion than among those receiving programmed intermittent epidural bolus analgesia in the postoperative period^10,11^. Unless there is a significant difference in the risk of elevating intracranial pressure between the two modes, the use of programmed intermittent epidural bolus may be preferred because it results in fewer side effects.

Padayachy *et al*. found that an optic nerve sheath diameter >5.75 mm has the best diagnostic accuracy for detecting an intracranial pressure >20 mmHg in children >1 year of age^31^. Compared with this threshold, the baseline values of optic nerve sheath diameter in some subjects of our study were quite elevated. However, the median age of the previous study population was 36 months and the mean age of subjects in our study was 9 years. This is important to note because between the ages of four and five, the optic nerve sheath grows to its adult size^27^. In a paediatric population with a similar mean age as in our study, the optic nerve sheath diameter measured using ultrasonography was 5.75 ± 0.52 (mean ± SD)^32^, which was comparable to the baseline optic nerve sheath diameter observed in our study population.

The limitations of this study are as follows. First, we observed the optic nerve sheath diameter for up to 70 min after applying the two epidural analgesia modes for 1 h. Thus, it is difficult to predict whether optic nerve sheath diameter continues to rise after the 70-min period observed in this study. Further research is needed to investigate how optic nerve sheath diameter changes after continuous application of both delivery modes. Second, the children who were included as subjects were undergoing osteotomy of the legs caused by cerebral palsy. However, we excluded patients with brain lesions or a history of increased intracranial pressure. Normal intelligence occurs in >60% of children with hemiplegia^8^, and the baseline optic nerve sheath diameter observed in our study was comparable to the distribution of optic nerve sheath diameter in unaffected children^32^. Thus, the results of this study are expected to be similar to those of the general paediatric population. Third, although the volume of saline administered was small (<2 ml), saline injected into the epidural space during loss of resistance confirmation could affect optic nerve sheath diameter.

In conclusion, although there were differences in the patterns of optic nerve sheath diameter change according to the lumbar epidural injection method, the bolus group was not associated with a greater risk of intracranial pressure increase than was the infusion group. Further research is needed to investigate intracranial pressure changes after continuous application of each delivery mode.

## Methods

### Patients

The Severance Hospital institutional review board (protocol number: 4-2017-0341) approved the study protocol (ClinicalTrial.gov, NCT03200951, 27/06/2017). This study was performed in accordance with relevant guidelines and regulations. Written informed consent was obtained from the children who could express consent and from the parents of all children. Forty patients (aged 4–14 years) among children undergoing lower extremity orthopaedic surgery at Severance Hospital who had body weights ≤40 kg and who had treatment plans for epidural analgesia were enrolled. The body weight limit was set to reduce variations in patient physique. Patients were excluded from the study if they had one or more symptoms or clinical signs of spinal anomalies or infection, coagulopathy, ophthalmic disease, increased intracranial pressure (baseline optic nerve sheath diameter >6.27 mm)^32^, history of increased intracranial pressure (e.g., ventriculoperitoneal shunt, hydrocephalus) or an expected duration of anaesthesia <70 min.

### Anaesthesia

Anaesthesia was induced with propofol (Fresofol 1% MCT, 2 mg·kg^−1^; Fresenius Kabi Austria GmbH, Graz, Austria), rocuronium (Rocumeron, 0.8 mg·kg^−1^; Ilsung Pharmaceuticals Co., Ltd., Seoul, Korea), and remifentanil (Ultian, 0.5–1 µg·kg^−1^; Hanlim Pharm. Co., Ltd., Seoul, Korea). After intubation, anaesthesia was maintained using sevoflurane in oxygen/air (40%) and remifentanil (0.05–0.15 µg·kg^−1^·min^−1^) to keep the bispectral index level within 40–60. All patients were mechanically ventilated in order to maintain 35–40 mmHg of end-tidal carbon dioxide (EtCO_2_) at a tidal volume of 8 ml∙kg^−1^, and a positive end-expiratory pressure of 3 cmH_2_O was applied.

A radial artery catheter and a peripheral intravenous line were placed after induction. Pulse oximetry, electrocardiography, gas analysis, invasive arterial blood pressure, oropharyngeal temperature, bispectral index, and EtCO_2_ concentration were used as standard monitors.

### Assignments and interventions

The surgeons, patients, and anaesthesiologist responsible for optic nerve sheath diameter measurement were all blinded to the group assignments during the entire study period. A computer-generated randomisation table (http://www.random.org) was used to assign 40 patients to the epidural bolus or infusion groups. The principal investigator (Y.S.C.) performed the randomisation and ensured the correct assignment for each patient. In the bolus group, the pump (Accumate 1200 v2.31, Woo Young Medical Co., Ltd., Seoul, Korea) was programmed to deliver 0.3 ml·kg^−1^ of 0.15% ropivacaine at a rate of 250 ml·hr^−1^. In the infusion group, the pump was programmed to infuse 0.15% ropivacaine at a rate of 0.3 ml·kg^−1^·hr^−1^. In both groups, the pumps were stopped 1 h after epidural analgesia. Ultrasonography (SonoSite X-PORTE, FujiFilm SonoSite Korea Ltd., Seoul, Korea) was used to visualise the lumbar vertebrae and epidural space after induction of anaesthesia. The skin-to-epidural space depth was measured with each patient in a left lateral decubitus position. An 18-gauge Tuohy needle was inserted at the L2–3 interspace level, and the epidural space was identified by loss of resistance to saline. A 20-gauge epidural catheter was inserted 3 cm upward into the epidural space. The possibility of intravascular placement was excluded using an aspiration test. The principle investigator set up the epidural pumps according to each patient’s assigned group. The pump was concealed in an opaque bag until the optic nerve sheath diameter measurements were completed.

### Measurement

**Figure 3:**
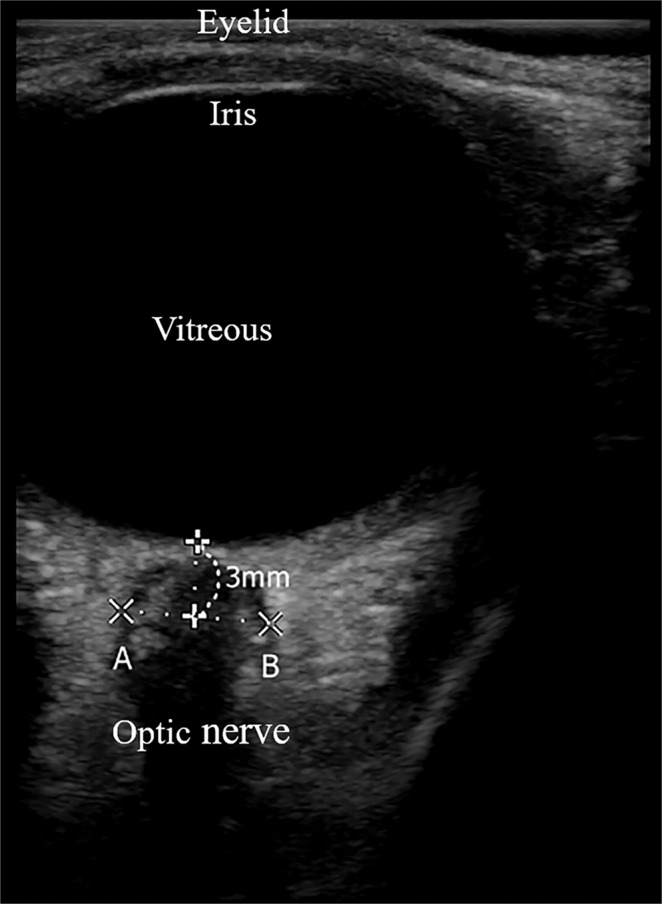
Measurement of optic nerve sheath diameter using ultrasonography. Axial plane images of the orbit were acquired through the optic nerve. Optic nerve sheath diameters were measured 3 mm posterior to the globe (**A**,**B**).

One investigator (B.L) with experience in over 50 cases of ultrasonographic optic nerve sheath diameter measurement used transorbital sonography to measure optic nerve sheath diameter; this investigator was blinded to group assignment. To reduce the risk of ultrasonic energy-induced eye injury, the ultrasonography was performed using a linear 6- to 13-MHz probe (FujiFilm SonoSite Korea Ltd., Seoul, Korea) with reduced power output (mechanical index = 0.2; thermal index = 0). After application of a thick sterile coupling gel layer to the closed upper eyelid, the probe was placed without exerting pressure. Axial images of the orbit were acquired in the plane of the optic nerve, and optic nerve sheath diameter was measured 3 mm posterior to the globe as described previously (Fig. 3)^15^. A 3.0–3.5 cm required depth was usually used. The optic nerve sheath diameters were measured at four time points: before starting the pump (T0) and 3 min (T1), 10 min (T2), and 70 min (T3) after starting the pump. Two measurements of each optic nerve sheath were acquired for each eye at each time point and these were averaged to obtain the optic nerve sheath diameter for that time point. Heart rate, invasive arterial blood pressure, end-tidal carbon dioxide concentration, end-tidal sevoflurane concentrations, and peak inspiratory pressure were recorded at each time point.

### Statistical analyses

Between-group differences in optic nerve sheath diameter were analysed. Based on previous findings, we considered a difference in optic nerve sheath diameter >0.4 mm (10% of mean optic nerve sheath diameter in paediatric subjects under general anaesthesia [4.3 ± 0.3 mm])^25^ to be clinically relevant. Twelve subjects were required for each group at a significance level of 5% and power of 90%. We enrolled 20 patients per group to compensate for withdrawals and observational variation.

The results for continuous variables are presented as mean (standard deviation) or median (interquartile range). The results for categorical variables are presented as numbers. Parametric data were analysed using independent *t*-tests. Nonparametric data were analysed using the Mann-Whitney U test. Fisher’s exact tests or χ^2^ tests were used to evaluate categorical variables. Linear mixed models of the fixed and random effects between the two groups were used to examine the repeated optic nerve sheath diameter measurements. Intergroup comparisons of optic nerve sheath diameter changes over time included group-by-time interactions. Correlations between repeated measures were examined using an unstructured covariance matrix. The linear mixed models for optic nerve sheath diameter were adjusted for parameters that might affect optic nerve sheath diameter, intracranial pressure, or both (e.g., sex, age, height, weight, end-tidal carbon dioxide concentration, end-tidal sevoflurane concentration, and peak inspiratory pressure). Post-hoc analyses of optic nerve sheath diameter using the Bonferroni correction for multiple comparisons were also performed. Areas under the curve were calculated using the estimated mean (least squares mean) changes in optic nerve sheath diameter over time from the linear mixed models.

All statistical tests were two-tailed. P values <0.05 were considered to indicate statistically significant results. All analyses were performed using SPSS 23.0 (IBM Corp., Armonk, NY, USA) and SAS 9.4 (SAS Inc., Cary, NC, USA).

## Data Availability

The data that support the findings of this study are available from the corresponding author on reasonable request.
